# Acute Kidney Injury in the ICU during Ganciclovir Treatment, an Observational Study

**DOI:** 10.3390/jcm12051898

**Published:** 2023-02-28

**Authors:** Mina Al Fartousi, Kaylen Guda, Tjerk H. Geersing, Peter H. J. van der Voort, Eric J. F. Franssen

**Affiliations:** 1 Department of Clinical Pharmacy, OLVG Hospital, 1091 AC Amsterdam, The Netherlands; 2 Department of Critical Care, OLVG Hospital, 1091 AC Amsterdam, The Netherlands; 3 Department of Critical Care Medicine, University Medical Center Groningen, University of Groningen, 9713 GZ Groningen, The Netherlands; 4 TIAS School for Business and Society, Tilburg University, 5037 AB Tilburg, The Netherlands

**Keywords:** ganciclovir, ICU, therapeutic drug monitoring, acute kidney injury, cytomegalovirus

## Abstract

The aim of this study is to investigate the relationship between ganciclovir exposure with TDM and the development of AKI in ICU patients. This retrospective single-center observational cohort study included adult ICU patients treated with ganciclovir who had a minimum of one ganciclovir trough serum level. Patients receiving less than two days of treatment and patients with fewer than two measurements of serum creatinine, RIFLE scores, and/or renal SOFA scores were excluded. Acute kidney injury incidence was assessed with the difference between the final and first values of the renal SOFA score, RIFLE score, and serum creatinine. Nonparametric statistical tests were performed. In addition, the clinical relevance of these results was evaluated. A total of 64 patients were included with a median cumulative dose of 3150 mg. The mean difference in serum creatinine during ganciclovir treatment was reduced by 7.3 μmol/L (*p* = 0.143). The RIFLE score decreased by 0.04 (*p* = 0.912), and the renal SOFA score was reduced by 0.07 (*p* = 0.551). This single-center observational cohort study showed that ICU patients using ganciclovir with TDM-guided dosing did not develop acute kidney injury as measured by serum creatinine, RIFLE score, and renal SOFA score.

## 1. Introduction

Cytomegalovirus (CMV) is a common virus, and CMV infections occur all over the world. In women of reproductive age, seroprevalence ranges from 45% to 100% [1]. Previous studies found a CMV infection rate of 17% in critically ill nonimmunocompromised patients, up to 30% in hematopoietic stem cell transplant recipients, and up to 60% in solid organ transplant recipients [2,3]. After primary infection, the virus remains latent in the body. Reactivation of the infection with renewed virus excretion is common in critically ill patients. In healthy patients with good immunity, this is asymptomatic [2,3]. A higher risk of complications is mostly found in congenital infections or in immunocompromised patients. Ganciclovir, an antiviral drug, is the drug of choice for prophylaxis and first-line treatment of CMV. As a group, antiviral drugs are associated with crystal nephropathy [4]. Moreover, ICU patients are at risk for multiorgan failure (MOF), and MOF has been identified as a risk factor for ganciclovir-induced toxicity [5]. Ganciclovir has a narrow therapeutic window [6]. Therapeutic drug monitoring (TDM) is used to ensure patient safety. The aim of TDM-guided dosing is to maximize effectiveness and minimize toxicity. Ritchie et al. investigated the association between serum ganciclovir levels, wherein doses were guided by TDM and ganciclovir efficacy and safety. Nephrotoxicity occurred in 45.1% of the patients [7].

Thus far, ganciclovir-induced nephrotoxicity has not been studied in a population with only intensive care unit (ICU) patients. The overall incidence of acute kidney injury (AKI) in ICU patients ranges from 20% to 50%, with a lower incidence seen in elective surgical patients and a higher incidence in sepsis patients [8]. Therefore, insight into the potential nephrotoxic effects of ganciclovir is important.

## 2. Materials and Methods

### 2.1. Aim, Design, and Setting

The aim of this study was to investigate the relationship between ganciclovir exposure and the development of AKI in ICU patients. The study was approved by the Medical Ethics Assessment Committee (MEC) of the OLVG Hospital in Amsterdam (MEC no. 19.081). This was a retrospective single-center observational cohort study in the OLVG. The ICU is a 20-bedded mixed medical and surgical tertiary ICU. Adult ICU patients treated with intravenously administered ganciclovir between 2008 and 2018 were included. Other criterion for inclusion was availability of at least one trough serum concentration of ganciclovir. Patients receiving less than two days of ganciclovir treatment and patients with less than two measurements of serum creatinine, RIFLE scores, and/or renal Sequential Organ Failure Assessment (SOFA) scores were excluded.

The values of these scores on the first day of ganciclovir administration were used as a baseline. A margin of 3 days before or 3 days after the start or stop date of ganciclovir was used. If there was no date for which all three scores were available within the range, patients were excluded.

All data were extracted from the patient data monitoring system (PDMS) Metavision^®^ (iMDsoft, Düsseldorf, Germany). The baseline characteristics included age, sex, body weight, SOFA score, APACHE III and IV score, trough serum levels, duration of ganciclovir treatment, and overall length of stay. 

### 2.2. Indicators of AKI

#### 2.2.1. Serum Creatinine 

The normal range of serum creatinine is 60–110 μmol/L in adults. An increase of serum creatinine of 1.5 times the patient’s baseline value after drug administration (±3 days) is considered clinically relevant and in concordance with the RIFLE criteria.

#### 2.2.2. RIFLE Score 

The RIFLE classification stands for the increasing severity classes risk, injury, and failure, as well as the two outcome classes, loss and end-stage renal disease (ESRD). The three severity grades are defined on the basis of the changes in serum creatinine or urine output where the worst of either criterion is used. The two outcome criteria, loss and ESRD, are defined by the duration of loss of kidney function [9,10]. The quantitative classification 0 was used for patients with no increase in serum creatinine or with an increase < ×1.5 or a decrease in GFR < 25%. Patients with renal replacement therapy were categorized as 3 in accordance with ‘haemodialysis’ (Table 1).

The RIFLE diagnosis and classification of AKI are displayed in Table 1. 

To compare the first and final RIFLE scores, the quantitative classification was used. A difference between first and final value of ≥1 is defined as clinically relevant. 

#### 2.2.3. Renal SOFA Score

The SOFA score is used as a follow-up score for daily assessment of patient organ failure during an ICU stay. This score is based on six different scores: one each for the respiratory, cardiovascular, hepatic, coagulation, renal, and neurological systems. The renal SOFA score is determined by serum creatinine and urine output.

The renal SOFA score classification is displayed in Table 2. 

In this study, the difference between the first and final value in renal SOFA score of ≥1 is defined as clinically relevant. 

### 2.3. Endpoints

The primary endpoint was the development of AKI during ganciclovir administration. AKI was defined as an increase in serum creatinine of 1.5 times the patient’s baseline value after drug administration [8,11] or a difference between the first and final values of the RIFLE score or renal SOFA score of ≥1. These values were selected based on arbitrary grounds.

The secondary endpoint was to assess the impact of the cumulative ganciclovir dose and trough serum level on the development of AKI and to assess the absolute number of patients in this population who developed AKI.

### 2.4. Therapeutic Drug Monitoring of Ganciclovir; Bioanalysis and Targets

CMV-infected patients received a standard dose of 300 mg twice daily and intravenously (1 h infusion). The aim of TDM-guided dosing is to maximize effectiveness and minimize toxicity. Thus far, no generally accepted therapeutic or toxic ranges have been validated. TDM-guided dosing of ganciclovir was routinely used in these patients with a reference value for a trough level of 0.5–3.0 mg/L [6]. In cases in which the level was too low, the dose was increased. In cases in which the level was too high, the dose was reduced according to the decision of the attending physician and pharmacist. TDM was continued after adjusting the dose. 

### 2.5. Statistical Analysis

All quantitative data were analyzed using SPSS (v22, Inc. Chicago, IL, USA). Categorical variables were reported as counts and percentages. Continuous variables were described using the mean for normally distributed data and the median for other distributions. Descriptive statistics were used to describe the baseline statistics. In cases of normally distributed data, the mean and standard deviation (SD) were given. For other distributions, the median and interquartile range (IQR) or number and percentage were given. Nonparametric tests were used to determine the significance of the differences, as they can be used in normal and other distributions. Pearson’s correlations were calculated to statistically investigate the relationship between the cumulative ganciclovir dose and the development of AKI. A *p*-value of 0.05 was used for statistical significance.

### 2.6. Impact of Cumulative Ganciclovir Dose and Trough Serum Level on Development of AKI 

To investigate the relationship between the cumulative ganciclovir dose and the development of AKI, scatterplots were constructed showing the relationship between the difference in serum creatinine, RIFLE, and renal SOFA score and the total administered ganciclovir dose. 

The total cumulative ganciclovir dose was plotted against the changes in serum creatinine, RIFLE scores, and/or renal SOFA scores. Pearson’s correlations were calculated to statistically investigate the results.

### 2.7. Number of Patients in This Population Who Developed AKI

To assess the number of patients who developed AKI in our population, the individual values of serum creatinine, RIFLE, and renal SOFA scores were collected and evaluated before and after ganciclovir treatment. Patients with an increase in serum creatinine of 1.5 times the baseline or an increase in RIFLE or renal SOFA score ≥ 1 were considered to have developed AKI based on the abovementioned definition of AKI.

## 3. Results

**Figure 1:**
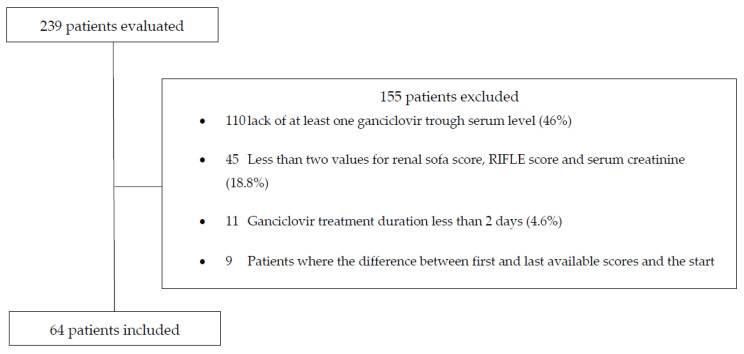
Patient enrolment.

A total of 239 patients received at least one dose of ganciclovir in the ICU between 2008 and 2018. A total of 175 patients were excluded for the reasons presented in Figure 1. Among the 64 included patients, the median age was 65.5 years, 38 (59.4%) were male, and the median weight was 76.5 kg. The patient baseline characteristics are presented in Table 3.

### 3.1. Development of AKI after Ganciclovir Treatment 

The mean serum creatinine during ganciclovir treatment was reduced by 7.3 μmol/L (*p* = 0.143). As shown in Table 4, the mean RIFLE score decreased by 0.04 (*p* = 0.912), and the mean renal SOFA score was reduced by 0.07 (*p* = 0.551). These differences were not significant nor clinically relevant. Table 5 and Table 6 show the distribution of the data for the RIFLE and renal SOFA scores.

### 3.2. Impact of Cumulative Ganciclovir Dose on Development of AKI 

**Figure 2:**
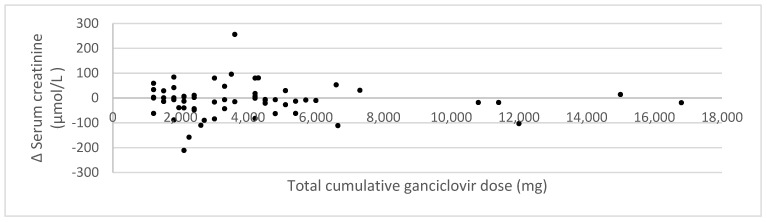
Impact of cumulative ganciclovir dose on the change in serum creatinine.

**Figure 3:**
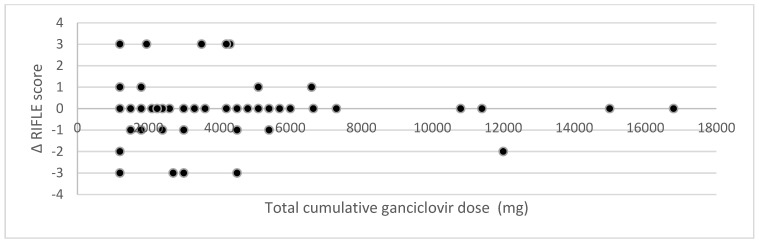
Impact of cumulative ganciclovir dose on the change in RIFLE score.

**Figure 4:**
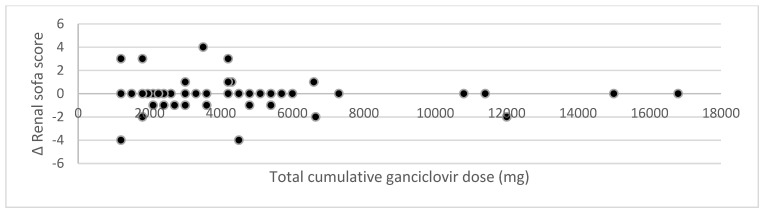
Impact of cumulative ganciclovir dose on the change in renal sofa score.

To investigate whether there is a relationship between the cumulative ganciclovir dose and the change in serum creatinine, RIFLE score, and renal SOFA score, scatterplots were constructed (Figure 2, Figure 3 and Figure 4). No relationship is visible. Statistical analysis through Pearson’s correlation coefficient confirmed the absence of a statistically significant relationship (*r* = −0.03 (*p* = 0.80); *r* = −0.07 (*p* = 0.61); *r* = −0.07 (*p* = 0.61) for changes in serum creatinine, RIFLE score, and renal SOFA score, respectively). 

### 3.3. Impact of Trough Serum Level on Development of AKI

The trough serum levels are, in most cases, within the therapeutic area, as we can see with the median and IQR of 1.3 (0.7–2.65). 

A total of 13 unique patients (20.3%) had an increase in serum creatinine of 1.5 times the baseline or an increase in RIFLE or renal SOFA score ≥1, and 7 of these patients had trough serum levels of ganciclovir higher than 3.0 mg/L.

## 4. Discussion

This study shows no indication of the development of AKI in ICU patients treated with ganciclovir and TDM. The mean values of serum creatinine, RIFLE, and renal SOFA scores did not change significantly, and the observed overall changes were not clinically relevant. 

Few studies have focused on the nephrotoxicity of ganciclovir, and they have reported different findings. Galar et al. showed that 4.3% of patients treated with ganciclovir or valganciclovir developed nephrotoxicity [9]. Ritchie et al. showed that 45% of patients treated with ganciclovir developed nephrotoxicity. In our population, 13 patients (20.3%) developed AKI based on our definition. Compared with the overall incidence of AKI in the ICU (20–50%), we did not observe a relationship between ganciclovir use and the development of AKI [8]. 

There are explanations for these differences. Our population consisted entirely of patients admitted to the ICU. Galar et al.’s population consisted of 21% ICU patients, and Ritchie et al.’s population consisted of 51% ICU patients. In addition, the definition of AKI used by Galar et al. and Ritchie et al. is not identical to the definition of AKI in our study. 

Multiple definitions have been used in the literature for AKI in critically ill patients, making comparisons difficult [11,12]. Galar et al. defined nephrotoxicity as an increase in serum creatinine during drug administration of 1.5 times the patient’s baseline value [9]. Ritchie et al. defined nephrotoxicity as an absolute increase in serum creatinine of greater than or equal to 0.3 mg/dL within 48 h or 1.5 times the patient’s baseline within seven days [7,11]. However, both Galar et al. and Ritchie et al. did not study urine output. 

Our study has several strengths and limitations. The strength of this study is that it is the first article that focuses on the potential nephrotoxicity of ganciclovir in critically ill patients. Another strength is that we used multiple scores to define AKI. In addition, TDM was used to guide the individual doses of ganciclovir. A weakness of this study is the relatively small number of patients, even though this is in accordance with previous studies [7,9]. Moreover, a large number of patients were excluded, which lowers the external validity of our study. The reason was missing data: lack of ganciclovir trough serum level, renal sofa score, RIFLE score, and serum creatinine or registered treatment time of fewer than two days. 

Not all the values (serum creatinine, RIFLE, and renal SOFA score) were available on the start and final day of ganciclovir treatment. A margin of 3 days before or 3 days after the start or stop date of ganciclovir was used. We accepted this margin of 3 days due to the retrospective nature of this study. Other nephrotoxic drugs were not excluded. Any nephrotoxicity could be due to the use of other drugs that are nephrotoxic. A randomized controlled trial is the preferred way to study the effects of TDM-guided dosing of ganciclovir on renal function. However, the present retrospective data provided the best available evidence.

## 5. Conclusions

In conclusion, this study of 64 critically ill patients treated with intravenously administered and TDM-guided ganciclovir treatment shows no indication of the development of clinically relevant AKI.

## Figures and Tables

**Table 1:** The RIFLE diagnosis and classification of AKI.

Class	Quantitative Classification	Serum Creatinine	Glomerular Filtration Rate (GFR)	Urine Output
Risk	1	Increase ×1.5	Decrease > 25%	<0.5 mL/kg/h for at least 6 h
Injury	2	Increase ×2	Decrease > 50%	<0.5 mL/kg/h for at least 12 h
Failure	3	Increase ×3 or >4 mg/dL (>354 µmol/L) with an acute rise > 0.5 mg/dL (>44 µmol/L) Or CVVHD/haemodialysis	Decrease > 75%	<0.3 mL/kg/h for at least 24 h or anuria for at least 12 h
Loss	4	Persistent acute renal failure = complete loss of kidney function > 4 weeks		
End-stage kidney disease	5	ESRD > 3 months		

**Table 2:** The renal SOFA score classification.

Renal SOFA Score	Serum Creatinine (mg/dL) [μmol/L]	Urine Output (mL/d)
0	<1.2 [<110]	
1	1.2–1.9 [110–170]	
2	2.0–3.4 [171–299]	
3	3.5–4.9 [300–440]	<500
4	>5.0 [>440] or hemodialysis	<200

**Table 3:** Baseline characteristics: data shown as median (IQR) or number and percentage.

Included Patients	ICU Patients Treated with Ganciclovir (*n* = 64)	
Age	65.5 (58–71)	
Male	38 (59.4%)	
Weight (kg)	76.5 (65–90)	
SOFA score	6 (5–8.75)	
APACHE III score	90 (80–100.5)	
APACHE IV predicted mortality	0.50 (0.31–0.69)	
Total amount of ganciclovir (mg)	3150 (1987.5–4725)	
Overall median ganciclovir daily dose (mg)	493 (348–600)	
Length of stay (days)	19 (13–34.5)	
Ganciclovir treatment duration (days)	7 (5–10)	
Trough serum levels (mg/L)	1.3 (0.7–2.65); overall range (0.1–13)	
Diagnosis	27; 42.2%	Pneumonia
	15; 23.4%	Other lung diseases
	11; 17.2%	Sepsis and septic shock
	6; 9.4%	Other
	5; 7.8%	Cardiac diseases

**Table 4:** Development of AKI during ganciclovir treatment, determined by the difference in RIFLE score, renal SOFA score, and serum creatinine, between the first and last day of treatment with ganciclovir.

	Score at the Beginning of Treatment (Mean ± SD)	Score at End of Treatment (Mean ± SD)	Difference between Final and Starting Value	*p*-Value *
Serum creatinine	94.5 ± 55.4	87.2 ± 54.2	−7.3	0.143
RIFLE score	0.75 ± 1.17	0.71 ± 1.16	−0.04	0.912
Renal SOFA score	0.73 ± 1.29	0.66 ± 1.32	−0.07	0.551

* Wilcoxon signed rank test.

**Table 5:** Distribution of RIFLE scores.

RIFLE Score	Beginning of Treatment (Number of Patients)	End of Treatment (Number of Patients)
Not applicable (0)	42	43
Risk (1)	7	8
Injury (2)	4	2
Failure (3)	11	11
Loss (4)	0	0
End-stage kidney disease (5)	0	0

**Table 6:** Distribution of renal SOFA scores.

Renal SOFA Score	Beginning of Treatment (Number of Patients)	End of Treatment (Number of Patients)
0	42	47
1	11	8
2	4	0
3	0	2
4	7	7
